# Preventive Dentistry: The Public Health Aspect

**Published:** 1922-11

**Authors:** Edwin N. Kent


### PREVENTIVE DENTISTRY: THE PUBLIC HEALTH ASPECT

BY EDWIN N. KENT, D. M. D.,

The fundamental object of all public health work is the prevention of disease.

Any organization of health workers, or any individual, who attempts to meet the general public welfare problems, connected directly or indirectly with the disease known as dental caries, likewise must realize that the prevention of this disease and not its cure is the main object. The great idea today in public health work is expressed in the rapidly extending establishment of health centers.

We are not instituted, primarily, to fight disease and repair its ruins; we are expected to go back to its starting point and take all steps in our power to assure the maintenance of public health. It is true that mouth hygiene public health workers are quite active in assisting in the establishment of public dental infirmaries, and this work will be necessary for some time to come, but it nevertheless seems quite evident that our intended mission can best be fulfilled if we confine most of our efforts to the field of Preventive Dentistry.

To successfully resist the onslaught of an enemy we must first have a knowledge of its offensive tactics.

What may we accept as a reasonable statement of the cause of dental caries?

In all of our public utterances we must be reasonable, not from the scientific viewpoint alone, but also from the viewpoint of the layman. It seems, from the evidence so far submitted, that we are safe in publicly expressing the theory that dental caries has a predisposing and an exciting cause.

The predisposing cause is malnutrition. Improper foods, deficient in toothbuilding ingredients, inhibit the physiological processes of construction and repair and lower the resistance of tooth structure.

The exciting cause is the corrosive action of an acid formed by the fermentation of food retained on or between the teeth.

To combat the predisposing cause we must advocate proper foods, and in our writings and talks to meet public requirements, such words as carbohydrates, proteins and bitamins are useless, if not actually harmful. We advise the generous use of milk, fresh fruit, vegetables, bread made from whole grain, cereals and eggs, and we spend little time in explaining just how these foods act to bring about the desired end.

We combat the exciting cause by advice under three heads:

1. Natures method of preserving mouth cleanliness, is accomplished through the excessive mastication of hard, resistant foods.

2. (And here we admit our weakness.) Two artificial methods of cleansing tooth surfaces are provided to meet prophylactic requirements brought about by our present-day dietetic habits—the tooth brush and floss silk.

3. Complete and effective prophylaxis, under existing conditions, also requires the thorough polishing of all tooth surfaces by a dentist or hygienist at regular intervals.

Obviously our main line of defense must be constructed in the field of education.

It has been said that ignorance is responsible for the present prevalence of dental caries. The medical and dental professions lack knowledge in many scientific fields which are important, directly and indirectly, in their influence on the progress of tooth destruction.

Diet is undoubtedly fundamental, but a proper dietetic program will not go far unless other factors that influence environment, mental attitude and properly functioning digestive organs, receive their due consideration.

Our public health work is greatly handicapped by the fact that manuscript coming from the pens of medical theorists is not censored. And it should be admitted, parenthetically, that authoritative censorship is not always available. Our endeavors, however, should be directed toward standardization if we would retain the confidence of the public. We should, above all things, never admit our weakness. In discussions such as these, where conflicting theories may be advanced, unedited quotations should not be allowed to reach the public press.

There is one rule of action that your essayist has always conscientiously followed and advocated in connection with public health work: "Never advance any theories or instruction that express a one-man idea." The majority is *sometimes* mistaken; the individual, *frequently*.

When a man writes or speaks to the public he should feel the full weight of his responsibility and express only proved facts. Our field of positive assertion is, admittedly, rather limited, but let us aim to keep within it, ever striving, however, to extend it by every means within our power.

I believe many of our research laboratories are dissipating their energies on matters of secondary importance, while they should be concentrating on the main problem.

When a pathogenic organism is launched into the circulatory stream at the end of a tooth root it is not as important to discover where its landing place may be as to ascertain what is back of the launching and see that other tooth root ends are not the scenes of like events in the future.

*We*, as a *profession*, need knowledge; knowledge of the active and contributing causes of dental caries and knowledge of more effective means of preventing the most prevalent of human diseases. But the results of scientific research come slowly and, perhaps, considering the fact that nearly all scientific investigators are self-appointed martyrs, serving for a remuneration that amounts to much less than that which they could easily command in commercial or professional fields, our progress to date is remarkable.

Much as our scientific ignorance inhibits our progress toward the prevention of dental disease, however, it does not concern the health worked as much as does the ignorance of the layman.

While it is a fact that we must know more of fundamental causes it is also a fact that our present knowledge is not effectively and extensively disseminated.

The general condition of the school children of this country, relative to mouth hygiene, is appalling, if not disgraceful.

Only 5 per cent. have clean and healthy mouths.

Scarcely 10 per cent. use a toothbrush daily.

The children in the first five grades in our public schools average six goodsized cavities.

The causes are many. Prominent among them are:

1st. Weak or inefficient organization of educational forces.

2d. Lack or absence of cooperation between health officials and practitioners.

3d. Incomplete standardization.

4th. Insufficient funds for public health work.

ORGANIZATION OF EDUCATIONAL FORCES.

1. Our educational forces, to be strong and effective, should be headed and directed by a central organization, sponsored by the National Dental Association and, preferably, having the cooperation and support of the Federal Government, the American Red Cross, the Russell Sage and Rockefeller Foundations, and other important organizations interested in the movement.

This central bureau, or parent organization, should be under the management of a carefully selected director, assisted by an advisory board of medical and educational specialists, its mission being the formulation of a standardized educational program that would reach all people effectively. Such an organization could easily eliminate much of the present wastes of time and energy dissipated in spasmodic local campaigns experimentally dealing with the problem in single communities.

We must, under existing conditions, however, deal with public health matters locally. Commonwealths and municipalities must have their local boards of health and each should have a representative from the dental profession.

Unless the entire medical profession is misled in its present reasoning and practice mouth disease is responsible for a large number of secondary ailments in other parts of the body and this is an important item in connection with public health. No board of health can be considered as properly organized to cope with the problems it is instituted to meet, unless it includes in its membership a representative of the special branch of medicine which deals with the most prevalent of all human diseases.

Many years ago, Sir William Osler, Professor Medicine, Johns Hopkins University, said, "In all the range of hygiene there is nothing more important than the hygiene of the mouth." Scientific findings since the day when this statement was made have increased the importance and significance of mouth hygiene a hundredfold.

But after the acquisition of a dental representative on the local health board there comes the necessity of a proper organization of his special forces.

This field of public health work seems to be a specialty in itself and not only the activities of the individual himself, but the checking up and support of his work by the entire profession is so important to the public, that it seems inconsistent that some provisions is not made in the curricula of our dental colleges for the proper instruction of students to assure an appreciation of their public obligations.

The average dentist seems to consider himself a thing apart from the general problem of public health. He can not dodge his public duties. There is no one man or group of men who can be held accountable. The burden rests upon us, as a profession, collectively. *We* are responsible for all public efforts to prevent the most prevalent of human diseases, and *we* are also responsible for the proper organization of the work.

### COOPERATION BETWEEN HEALTH OFFICIALS AND MEDICAL AND DENTAL PRACTITIONERS.

2. Cooperation between health officials and practitioners is an important item in public health work.

This can best be assured in our field by the appointment of men for State or municipal office who are selected by the

State dental association, or a district representation of that body. Political influence should be eliminated as far as possible. The further appointment of an advisory committee to act officially under the State society is also advisable. But back of the man and the advisory committee the cooperation of the entire profession is necessary.

On one man, alone, can conceive the ideas; can work out the details; can solve the problems which will bring large results in the prevention of dental disease. If he does his *best* he is limited by the ability of one man. No one man, independent of his fellows, is able to meet the requirements. Even a small group of men cannot be relied upon to express the consensus of opinion, unless they are in intimate contact with the rank and file of the specialists they represent.

But public health workers seek cooperation in another direction.

If you agree that the gospel of mouth hygiene should be preached to the people of your community set an example in your own office.

The public lecturer is not infrequently approached by members of his audience who complain because their dentists have not brought to their attention the facts he has presented. He should not be required to meet this embarrassing situation.

It seems to be generally conceded that approximately 50 per cent. of dental decay may be prevented by proper attention to the dental toilet.

A very large percentage of the average dentist's patients do not realize the importance of mouth cleanliness. They need instruction and it is reasonable for them to expect it from their dentists. No part of the dental practitioner's operative procedure is more important than his advice to his patients on the home care of the mouth.

To no small extent, also, the general effect of our present mouth hygiene campaign is dependent on the efforts of the practitioner in his direct contact with individuals, and he should realize his responsibility.

STANDARDIZATION OF PUBLIC HEALTH WORK.

3. Standardization in public health work is, to my mind, essential to success. We sit in session at dental society meetings and receive from the lips of numerous theorists entrancing theories. It is quite fitting and consistent that we should hear all sides of the story—all evidence in the case. But the public should never hear any expression other than the verdict. And the verdict represents our expression of standardization. Standardization is simply the consensus of opinion of a group of men who are individually qualified to express themselves on the subject in question.

As an example, we have many methods, or many slightly varying systems, of technique in the use of the toothbrush.

Suppose we appoint a committee of twenty men to decide upon a standard. We can not reasonably expect these twenty men to agree on the first ballot, so to speak. But if eight of them advocate a sweeping motion, eight a circulating or rotary motion, and four a crosswise motion, it is obvious that a reasonable decision might be a combination of the sweeping and rotary motions, and this may, for the present, be adopted as standard.

But (and here comes the important point) the defeated minority must accept the decision as standard. They must, each one, be willing to say, "I was wrong;" "I will henceforth advocate the procedure decided upon."

This requires a broadness of mind that we must admit is not universal, but, in my experience, it is frequently enough in evidence to indicate that we can reach a reasonable standardization if we organize with that end in view.

In our selection of instruments for public clinic equipment we have pursued this course with very favorable results. We have submitted to extraction specialists a list of forceps recommended for use on a certain tooth. If our selected group of specialists numbered twenty men and fifteen voted for a definite model of forceps, the matter was settled. No other type of instrument could reasonably be accepted for this particular use, no matter what I, or any other interested person thought about it.

Standardization in mouth hygiene instruction is absolutely necessary to hold the confidence of the public, and a whole evening could be devoted to this one branch of the subject, I believe, with great advantage to public welfare.

PROPER REMUNERATION FOR HEALTH WORKERS.

4. I approach the subject of insufficient funds with some hesitation, mainly because I sincerely believe it is the most serious question we have to deal with in connection with the general consideration of public health problems.

I think I shall meet no opposition when I say that the type of man who can produce results in public health work in our special field is a man of specialized and, perhaps, unusual ability. He must have high professional standing; he must have sufficient scientific knowledge; he must be efficient as an organizer. Such a man usually commands more than the average income in private practice.

If we choose to put the matter in other words, such a man is worth more to the public than the average man earns in private practice. Perhaps it is reasonable to say that if he successfully works out the problem from national headquarters he is *worth* more than the highest remuneration coming to *any* dentist in private practice. It is plainly evident that we should pay such a man what he is worth. This, under present conditions, is impossible.

Our financial problem in Massachusetts is not materially different from that in other States. The present yearly salary of the Commissioner of Public Health is ($7500) seventy-five hundred dollars. In the epidemic of influenza a few years ago the fatalities outnumbered those who met death in the face of German guns in the World War.

Our Commissioner of Public Health is the commanding officer who plans our defense against these enemies within our lines, who are proving and have always proved more destructive and dangerous than any that have ever arisen from without.

Now, our great object is admittedly the prevention of disease, and, therefore, the so-called Division of Hygiene under the State Health Department must be considered as representing its most important branch of service. The man who presides over this branch of service; who conceives and executes the plans for the maintenance of public health receives the princely salary of ($4000) four thousand dollars per annum.

If we had a subdivision of Mouth Hygiene it would come under the Division of Hygiene and a man selected to take charge of such work could not be expected to receive a salary to equal more than 75 per cent. of that of his superior officer. It is probable that if such an office were created the powers that be would limit the salary to about ($2500) twenty-five hundred dollars.

It must be evident that the type of man who is big enough to fill such an office would be required to make a very large financial sacrifice in accepting the commission, as he could earn much more in private practice. Occasionally we find a man who is so situated financially that he does not need to consider income in connection with his life-work, or one whose altruistic spirit prompts him to sacrifice personal and family interests to the interests of the public. Under present conditions, from these two classes we must, as a rule, select our efficient public health officials.

I am not prepared to suggest a definite scheme for the solution of the problem, but I believe that, before we accomplish extensive results in the prevention of dental caries, it must be solved. Adequate funds must be provided to meet all necessary expense for campaigns of education and the follow-up work needed to assure that all of our people receive practical instruction on the value of a clean and healthy mouth.

There is no one that I know of in this country today who is directing state or municipal public health work on mouth hygiene who is not doing so at great personal sacrifice. With very few exceptions they are serving without remuneration, most of them lone missionaries in a cause which they hope to develop to the point where its importance will be recognized and they may relinquish their offices to a successor who will be appointed for full time service.

If preventive dentistry is worth while, its promulgation by state and municipal health departments is an important part of their work and we should determine the expense necessary to pursue the logical solution of the educational problem and insist that such appropriation be made as will meet the requirements.

In conclusion, the great need of the public in connection with the maintenance of oral health is education. It can be accomplished through a strong continuation of educational forces acting under a national organization with the enthusiastic co-operation of practitioners.

The whole educational scheme should be properly standardized by selected specialists, and last, but not least, an appropriation of funds should be insisted upon that will assure for all of our citizens the instruction they need to convince them of the logic and the importance of preventive dentistry.—*Dental Cosmos*, June, 1922.

